# Measuring the Impact of COVID-19 on Siyan Mental Health Patients Using the Epidemic-Pandemic Impacts Inventory: Survey Study

**DOI:** 10.2196/29952

**Published:** 2021-07-29

**Authors:** Anish Shah, Michele Darling, Olivia Arstein-Kerslake, Tiffany Morgan, Aubreen Vance Tovrea, James Young, Helen Laines

**Affiliations:** 1 Siyan Clinical Corporation Santa Rosa, CA United States; 2 LPC Consulting Associates Sacramento, CA United States

**Keywords:** COVID-19, coronavirus, pandemic, mental health, social isolation, wellness

## Abstract

**Background:**

Recent research has shown that the impacts of the COVID-19 pandemic and social isolation on people’s mental health are quite extensive, but there are limited studies on the effects of the pandemic on patients with mental health disorders.

**Objective:**

The objective of this study was to assess the negative impacts of the COVID-19 pandemic on individuals who have previously sought treatment for a mental health disorder.

**Methods:**

This study uses the newly developed Epidemic-Pandemic Impacts Inventory (EPII) survey. This tool was designed to assess tangible impacts of epidemics and pandemics across personal and social life domains. From November 9, 2020, to February 18, 2021, a total of 245 adults recruited from a mental health clinic completed the consent form and responded to the survey link from the Siyan Clinical Corporation and Siyan Clinical Research practices located in Santa Rosa, California, USA.

**Results:**

We found that the least affected age group included individuals aged 75 years or older. This was followed closely by the 65- to 75-year-old age group. People with children under the age of 18 years also reported both more negative indicators associated with the pandemic and more positive indicators compared to those without children at home. Gender queer, nonconforming, and transgender individuals may also be at higher risk for more negative impacts associated with the pandemic. When respondents were assessed with regard to their mental health diagnosis, no differences were noted. Substance use also increased during the pandemic.

**Conclusions:**

In conclusion, the data collected here may serve as foundational research in the prevention, care, and treatment of mental health disorders during pandemics such as COVID-19. Populations such as those with previously diagnosed mental health disorders are particularly at risk for negative effects of pandemic-related stressors such as social isolation, especially if they have children in the household, are part of a younger age group, or have substance use disorder. Gender may also be a factor. Further, the EPII survey may prove to be a useful tool in understanding these effects. Overall, these data may be a critical step toward understanding the effects of the COVID-19 pandemic on populations with a mental health diagnosis, which may aid mental health practitioners in understanding the consequences of pandemics on their patients’ overall well-being.

**Trial Registration:**

ClinicalTrials.gov NCT04568135; https://clinicaltrials.gov/ct2/show/NCT04568135

## Introduction

The World Health Organization announced that the COVID-19 outbreak had become a global pandemic on March 11, 2020. One year later, approximately 29.5 million people within the United States alone had been infected with the COVID-19 virus [1]. To combat this deadly infection, countries such as the United States took actions including social distancing, mask mandates, closures of schools and universities, remote or home-based working, and travel restrictions. However, although effective from a public health perspective, the potential impacts of the pandemic and social isolation on people’s mental health and on the mental health care system are extensive.

Research published since the height of the COVID-19 pandemic demonstrates the deleterious effects of this event on psychological well-being. One cross-sectional study conducted across 34 hospitals in China demonstrated that among health care workers exposed to COVID-19, women, nurses, those in Wuhan, and frontline health care workers have a high risk of developing negative mental health outcomes and may need psychological support or interventions [2]. In the early stages of the COVID-19 pandemic, researchers in China observed that 53.8% of survey respondents rated the psychological impact of the outbreak as moderate or severe, 16.5% reported moderate to severe depressive symptoms, and 28.8% reported moderate to severe anxiety symptoms [3]. Finally, another study conducted in China of about 18,000 social media users and their online posts demonstrated that COVID-19 was associated with an increase in negative emotions, such as anxiety, depression, and anger, and a decrease in positive emotions and life satisfaction [4].

The effects of COVID-19 have been far-reaching beyond China, however, and the impact of the virus has been felt across the globe. Within the United States, a survey conducted during the last week of March 2020 showed that 72% of Americans felt that their lives were impacted by the outbreak, a 32% increase from the survey conducted only 2 weeks earlier [5]. In the United Kingdom, the prevalence of clinically significant levels of mental distress rose from 18.9% in 2018-2019 to 27.3% in April 2020, one month into their own lockdown; further, these changes in mental health were greatest among young adults (aged 18 to 34 years), women, and people living with young children [6]. Problems with psychological well-being were also observed in Australia. For instance, during the first month of the stage 2 COVID‐19 restrictions, mental health problems were widespread among Australians; in addition, about one‐quarter of survey respondents reported mild to moderate symptoms of depression or anxiety [7].

The primary goal of this study was to describe the impacts of COVID-19 on a population of residents in the United States who had previously sought psychiatric services at the Siyan Clinical Corporation and Siyan Clinical Research practices located in Santa Rosa, California. Our objective was to assess the impact of the current COVID-19 pandemic on participants with previously diagnosed psychiatric disorders to identify areas of needed support and services. We were interested in whether or not there were any differences in the impact of the COVID-19 pandemic based on features such as age, gender, and mental health diagnosis. The data collected may serve as foundational research in the prevention, care, and treatment of mental health disorders during pandemics such as COVID-19. We also used a newly developed tool, the Epidemic-Pandemic Impacts Inventory (EPII) survey, to assess tangible impacts of epidemics and pandemics across personal and social life domains.

## Methods

### Recruitment

Approximately 3500 adult patients in the Siyan Clinical Corporation and Siyan Clinical Research practices (Santa Rosa, California, USA) were invited to participate in a one-time anonymous survey to assess tangible impacts of epidemics and pandemics across personal and social life domains. Siyan staff derived a list of patients from the Siyan electronic health record system who met eligibility criteria and sent an email inviting them to participate in the survey, explaining the purpose of the survey, how Siyan will use the survey results, and how the survey will be administered. Interested patients signed and returned an informed consent form (ICF) and then received a link to the online survey. A total of 326 patients out of 3500 completed the consent form and received the survey link from Siyan, for a response rate of 9.3%. Of these 326 patients, a total of 245 people responded from November 9, 2020, to February 18, 2021, for a completion rate of 75.2% among those who had completed the consent form. Patients received a US $10 eGift card from Starbucks upon completion of the survey.

These procedures were reviewed by the Advarra Institutional Review Board (IRB) and informed consent was obtained from all participants. The protocol, ICFs, principal investigator curriculum vitae, and all subject-facing and recruitment materials were submitted to the Advarra IRB for review in June 2020. A notice of intent was sent to the authors of the EPII survey prior to IRB submission. The ICF collection process was outlined in the approved protocol and most patients signed the ICFs via Adobe Sign. The data were collected through an approved process utilizing the Alchemer cloud-based integrated feedback platform. In September 2020, the protocol was determined to be IRB exempt by Advarra. This study was conducted using ethical principles derived from international guidelines including the Declaration of Helsinki and the Council for International Organizations of Medical Sciences International Ethical Guidelines.

This study was reviewed and published on ClinicalTrials.gov (NCT04568135) on September 29, 2020.

### The Epidemic-Pandemic Impacts Inventory

The EPII is a newly developed, 92-item tool designed to determine the impacts of epidemics and pandemics in personal and social life domains developed by Grasso and colleagues [8,9]. The EPII is divided into 10 subcategories, with a varying number of indicators in each subcategory: work and employment, education and training, home life, social activities, economic activities, emotional health and well-being, physical health problems, physical distancing and quarantine, infection history, and positive change. All domains except for *positive change* indicate negative or adverse experiences. Respondents were presented with indicators in each subcategory and asked, “Since the coronavirus disease pandemic began, what has changed for you or your family?” Participants then responded with *yes, me;* *yes, person in home;* *no*; or *N/A* (not applicable).

### Inclusion and Exclusion Criteria

#### Inclusion Criteria

A participant had to meet the following criteria to be eligible to participate in this study:

1. Voluntarily agreed to participate in the study under their own free will and was willing and able to agree to an e-ICF indicating that he or she understood the purpose of the study, he or she understood the procedures that were required for the study, and that he or she was willing to participate in the study.
2. Was female or male and between the ages of 18 and 80 years, inclusive, at the time of consent.
3. Was receiving or had previously received psychiatric services from Siyan Clinical Corporation and/or Siyan Clinical Research practices.
4. Was capable of understanding and complying with study requirements.
5. Had agreed to the e-ICF. No study-related procedures would be performed before the participant had agreed to the consent letter.

#### Exclusion Criteria

A participant who met any of the following criteria were excluded from this study:

1. Had a known diagnosis of dementia.
2. Was under the age of 18 years or over the age of 80 years.

### Data Analysis

Following the analytic approach taken in a preliminary report [9] using the EPII, the two *yes* responses (*yes, me*; *yes, person in home*) were collapsed, as were the *no* and *N/A* responses, to create dichotomous indicators. For the sake of this analysis, the nine *negative* subcategories were combined to form an overall *negative impacts* category, reporting the average number of indicators with *yes* responses. Results from the subcategories were also reported. Exploratory statistical analyses have also been performed. One-way analysis of variance (ANOVA) followed by Tukey post hoc tests, Student *t* tests, or chi-square tests were used, where appropriate. Results are reported as mean (SD) throughout the text, while figures show mean ± standard error of the mean (SEM). Statistical significance was defined as *P*<.05.

## Results

### Sample Characteristics

Table 1 details the sociodemographic characteristics of the sample. Most of the sample self-identified as female (76.3%) and White (84.5%). About half (54.2%) of the sample reported being currently employed. The majority of the sample reported being diagnosed with mood disorders (76.3%) or anxiety disorders (76.3%). Note that individuals could select multiple mental health diagnoses on the survey. Additionally, nearly half (47.8%) of the sample reported earning a bachelor’s degree or higher. We also compared the age and gender distributions in our sample against the population that sample was drawn from in chi-square analyses. We found no differences in the expected distribution of the age of the subjects (*χ*^2^_2_=7.2, *P*=.33; N=245). However, we did find a significant effect of sex distribution between the sample and the population (*χ*^2^_6_=107.5, *P*<.001; N=245). This large effect was likely driven by the disproportionate number of women that completed the survey (women comprised 76.3% of the sample but only 57.9% of the population).

**Table 1:** Sociodemographic characteristics of the sample.

Variable		Respondents (N=245), n (%)
**Age (years)**		
	18 to 24	25 (10.2)
	25 to 34	39 (15.9)
	35 to 44	57 (23.3)
	45 to 54	40 (16.3)
	55 to 64	47 (19.2)
	65 to 74	24 (9.8)
	75 or older	13 (5.3)
**Gender**		
	Female	187 (76.3)
	Male	52 (21.2)
	Other (gender queer, nonconforming, or transgender)	6 (2.4)
**Ethnicity**		
	American Indian or Alaska Native	7 (2.1)
	Asian	5 (2.0)
	Black or African American	5 (2.0)
	Hispanic or Latino	24 (9.8)
	White	207 (84.5)
	Other	6 (2.4)
	Undisclosed	9 (3.7)
**Education**		
	Less than high school	3 (1.2)
	Graduated high school	20 (8.2)
	Trade or technical school	8 (3.3)
	Some college, no degree	62 (25.3)
	Associate degree	34 (13.9)
	Bachelor's degree	74 (30.2)
	Advanced degree (master's degree, PhD, or MD)	43 (17.6)
	Undisclosed	1 (0.4)
**Children under 18 years of age living at home**		
	Yes	80 (32.7)
	No	162 (66.1)
	Undisclosed	3 (1.2)
**Employment status**		
	Employed, full time	98 (40.0)
	Employed, part time	35 (14.3)
	Unemployed, disabled	22 (9.0)
	Unemployed, looking for work	19 (7.8)
	Unemployed, not looking for work	6 (2.4)
	Unemployed, retired	32 (13.1)
	Unemployed, volunteer work	1 (0.4)
	Other—write in	29 (11.8)
	Undisclosed	3 (1.2)
**Mental health diagnosis**		
	Anxiety disorders	187 (76.3)
	Eating disorders	50 (20.4)
	Mood disorders	187 (76.3)
	Personality disorders	8 (3.3)
	Psychotic disorders	2 (0.8)
	Substance abuse disorders	29 (11.8)
	Trauma-related disorders	75 (30.6)
	Other	19 (7.8)
	Undisclosed	7 (2.9)
**Marital status**		
	Divorced	33 (13.5)
	Married or domestic partner	135 (55.1)
	Separated	7 (2.9)
	Single or never married	58 (23.7)
	Widowed	9 (3.7)
	Other	1 (0.4)
	Undisclosed	1 (0.4)

### Overall Analysis

On average, respondents selected *yes* to 22.96 (SD 8.75) negative indicators, out of a total of 73 negative indicators. Respondents selected *yes* to 6.76 (SD 3.61) positive indicators on average, out of a total of 19 positive indicators.

#### Age

**Figure 1:**
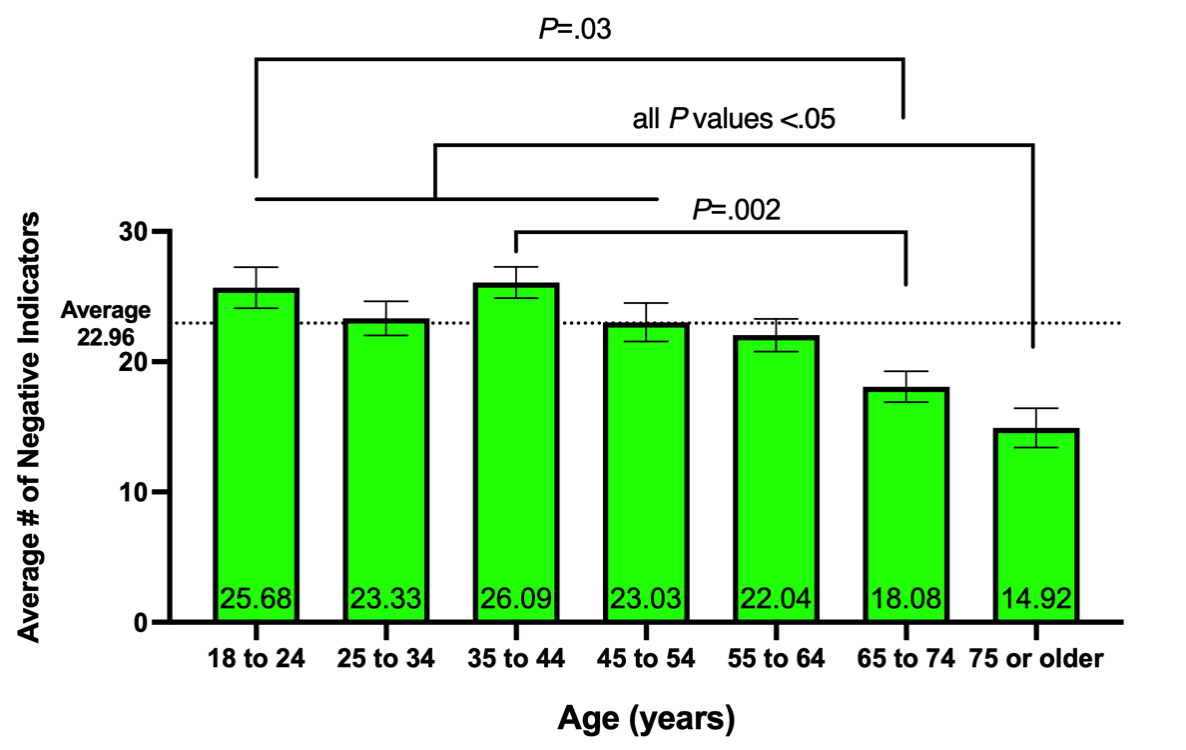
Average number of negative indicators by age. Respondents who were 75 years of age or older reported the lowest number of negative indicators (mean 14.92) on the Epidemic-Pandemic Impacts Inventory. The 65- to 75-year-old respondents reported the next lowest number of negative indicators (mean 18.08). Results are shown as mean (displayed on the bars) ± standard error of the mean (whiskers). *P* values were derived from a Tukey post hoc test.

Overall, there was a significant effect of age (*F*_6, 238_= 5.292, *P*<.001) (Figure 1). The 75-years-or-older age group (n=13) had the lowest number of negative indicators, with an average of 14.92 (SD 5.45) across all negative categories (ie, work and employment, education and training, home life, social activities, economic activities, emotional health and well-being, physical health problems, physical distancing and quarantine, and infection history). Tukey post hoc comparisons showed that the 75-years-or-older age group had significantly fewer negative indicators compared to the 18- to 24-year-old age group (*P*=.004), the 25- to 34-year-old age group (*P*=.03), the 35- to 44-year-old age group (*P*<.001), and the 45- to 54-year-old age group (*P*=.04). The 65- to 75-year-old age group (n=24) reported the next lowest number of negative indicators, with an average of 18.08 (SD 5.83) negative indicators. Specifically, they reported fewer negative indicators compared to the 18- to 24-year-old age group (*P*=.03) and the 35- to 44-year-old age group (*P*=.002).

#### Gender

**Figure 2:**
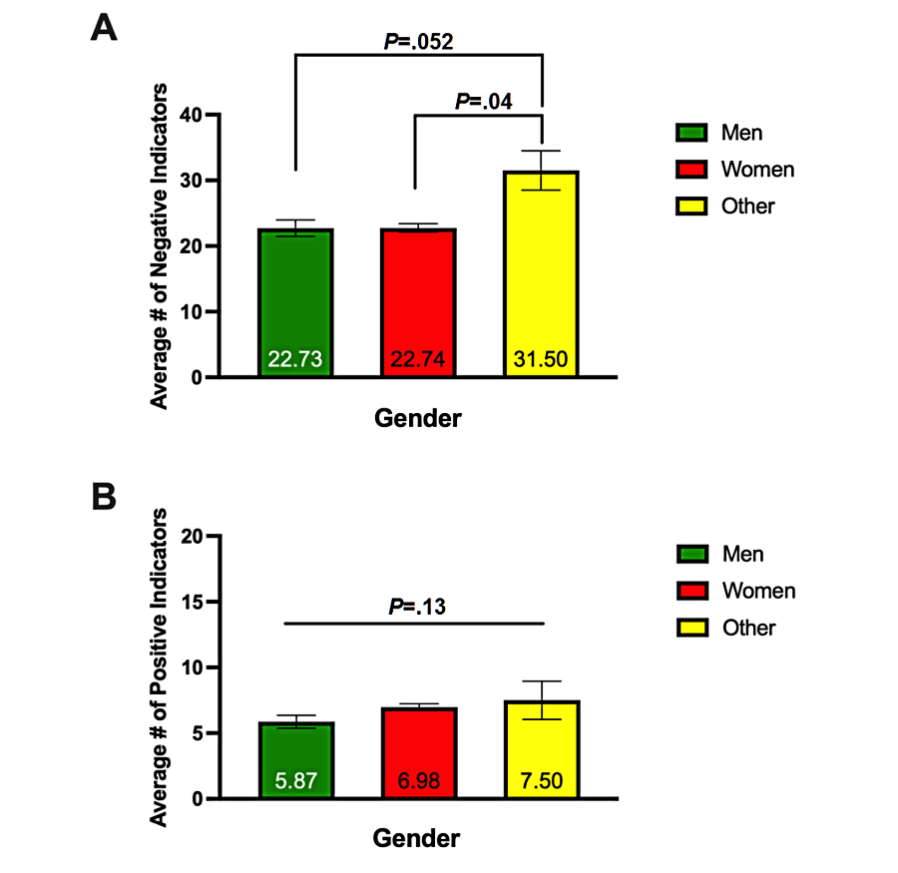
Negative (A) and positive (B) indicators were analyzed as a function of gender. Gender queer, nonconforming, and transgender individuals reported more negative indicators than men and women, on average. Results are reported as mean (displayed on the bars) ± standard error of the mean (whiskers). *P* values are overlaid on the figure.

We also analyzed the reported number of negative and positive indicators as a function of gender. In a one-way ANOVA analysis, the average negative indicators trended toward significance between men, women, and other gender queer, nonconforming, or transgender individuals (*F*_2, 242_=2.980, *P*=.053) (Figure 2A). Tukey post hoc comparisons showed that men tended to report fewer negative indicators than gender queer, nonconforming, or transgender individuals (*P*=.052), while women also reported significantly fewer negative indicators than this group (*P*=.04). There was a nonsignificant effect of positive indicators across genders (*F*_2, 242_=2.073, *P*=.13) (Figure 2B).

#### Stay at Home

An additional topic of interest was whether there would be any differences between respondents who completed the survey before, during, or after the stay-at-home order. Time frames aligned with the dates when the California regional stay-at-home order was implemented (December 3, 2020) and lifted (January 25, 2021). Therefore, the time frames before, during, and after the stay-at-home order were defined as follows, and surveys were aggregated along these dates:

1. Pre–stay-at-home order: surveys completed before December 3, 2020.
2. During stay-at-home order: surveys completed from December 4, 2020, to January 25, 2021.
3. Post–stay-at-home order: surveys completed on January 26, 2021, or after.

It is relevant to note that the sample size for the pre–stay-at-home order group was much smaller (n=17) than that of the during stay-at-home order group (n=135) and the post–stay-at-home order group (n=93). There were minimal differences across groups: the average number of negative indicators was 22.53 (SD 7.04) for the pre–stay-at-home order group, 23.3 (SD 8.93) for the during stay-at-home order group, and 22.54 (SD 8.83) for the post–stay-at-home order group (*F*_2, 242_=0.2281, *P*=.80). The average number of positive indicators was 5.06 (SD 3.05) for the pre–stay-at-home order group, 7.07 (SD 3.89) for the during stay-at-home order group, and 6.61 (SD 3.20) for the post–stay-at-home order group (*F*_2, 242_=2.491, *P*=.09).

#### Having Children

**Figure 3:**
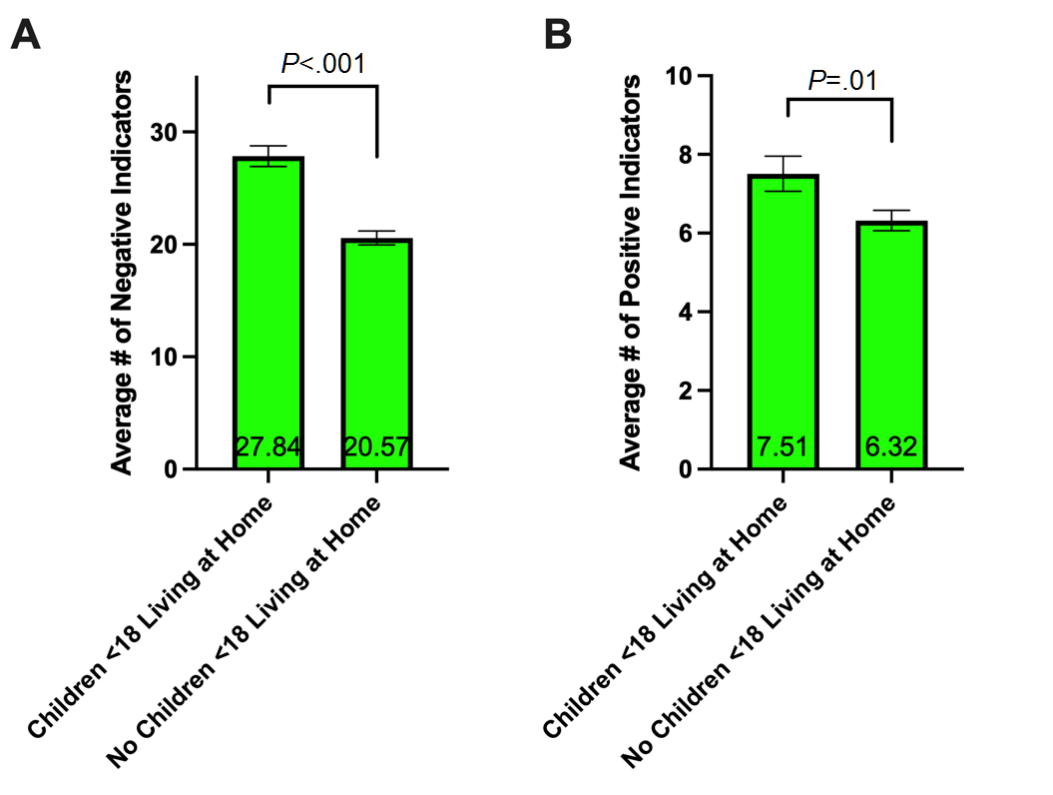
Average number of negative indicators (A) and positive indicators (B) as a function of having children living at home. Respondents with children under 18 years of age living at home (n=80) reported higher numbers of overall negative and positive impacts compared to respondents without children under 18 years of age living at home (n=162). Results are shown as mean (displayed on the bars) ± standard error of the mean (whiskers). *P* values were calculated from Student *t* tests.

Respondents with children under 18 years living at home (n=80) reported a significantly higher number of overall negative impacts (mean 27.84, SD 8.20), on average, compared to respondents without children under 18 years living at home (n=162) (mean 20.57, SD 7.95) (*t*_240_=6.623, *P*<.001) (Figure 3A). However, respondents with children under 18 years living at home also had a significantly higher number of positive impacts (mean 7.51, SD 4.00), on average, compared to respondents without children under 18 years living at home (mean 6.32, SD 3.28) (*t*_240_=2.465, *P*=.01) (Figure 3B). The difference between respondents with and without children was greatest in the *home life* subcategory, with an average number of impacts of 4.34 (SD 2.18) for respondents with children under 18 years living at home compared to 1.27 (SD 1.76) for respondents without children under 18 years living at home (*t*_240_=11.77, *P*<.001).

### Most Impacted Subcategories

#### Overview

The *social activities* subcategory showed the greatest impact from COVID-19. This category had 10 questions in total, with an average of 5.88 (SD 1.90) *yes, me* or *yes, person in home* responses. The next most impacted category was *emotional health and well-being*, with an average of 3.96 (SD 1.61) *yes* responses, followed by *physical health and well-being*, with an average of 3.93 (SD 1.55) *yes* responses.

#### Substance Abuse During COVID-19

About one-third (86/245, 35.1%) of the respondents reported an increase in the use of alcohol or substances.

#### Mental Health Diagnosis

**Figure 4:**
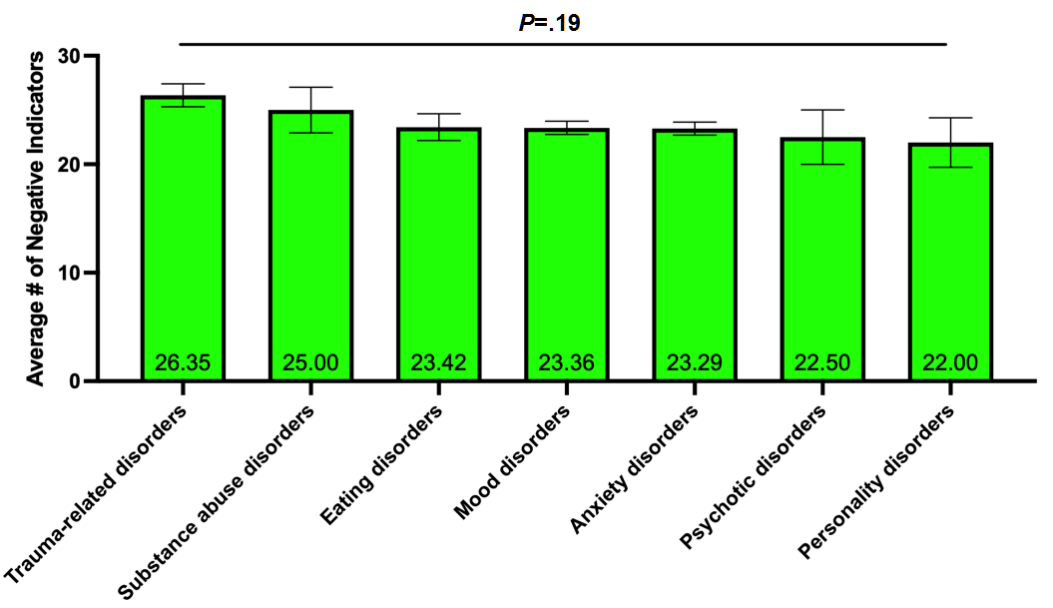
Average number of negative indicators as a function of mental health diagnosis. Results are shown as mean (displayed on the bars) ± standard error of the mean (whiskers).

The most prevailing *mental health diagnosis* in the data set was mood disorder (187/245, 76.3%) and anxiety disorders (187/245, 76.3%); the next most prevalent diagnosis was trauma-related disorder (75/245, 30.6%). However, there was no effect of mental health diagnosis on the number of negative indicators reported on the EPII (*F*_6, 531_=1.452, *P*=.19) (Figure 4).

#### Positive Change

**Figure 5:**
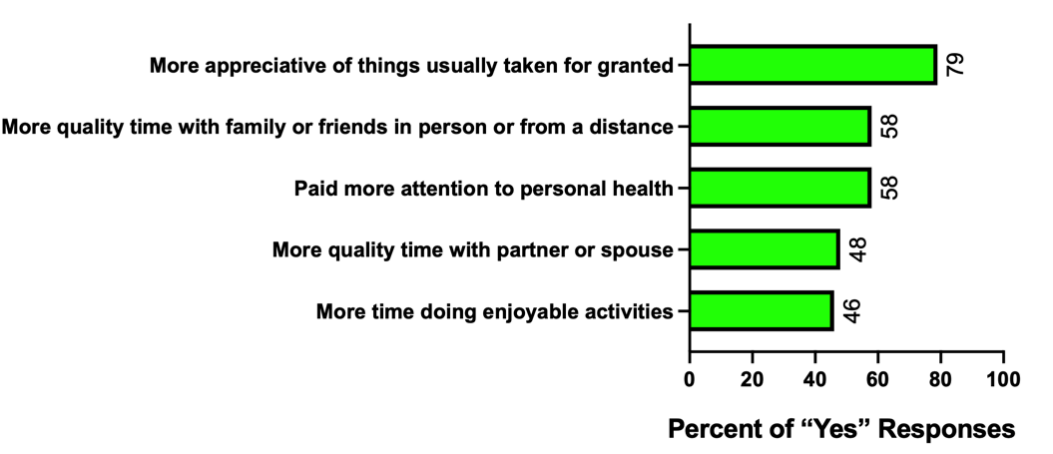
Top five positive indicators. The most reported indicator in positive change was "More appreciative of things usually taken for granted," followed by "More quality time with family or friends in person or from a distance".

The most reported indicator in *positive change* was “More appreciative of things usually taken for granted” (192/245, 78.4%), followed by “More quality time with family or friends in person or from a distance” (eg, on the phone, email, social media, video conferencing, and online gaming) (141/245, 57.6%) and “Paid more attention to personal health” (141/245, 57.6%) (Figure 5).

#### Most Impacted Indicators

Table 2 outlines the top two indicators in each subcategory with the highest percentage of respondents reporting *yes, me* or *yes, person in home*.

**Table 2:** The most impacted indicators.

Subcategory and survey items^a^		Respondents (N=245), n (%)^b^
**Work and employment**		
	Increase in workload or work responsibilities.	121 (49.4)
	Had to continue to work even though in close contact with people who might be infected (eg, customers, patients, and coworkers)	113 (46.1)
**Education and training (only two indicators in this subcategory)**		
	Had a child in the home who could not go to school	91 (37.1)
	Adult unable to go to school or training for weeks or had to withdraw	48 (19.6)
**Home life**		
	Increase in verbal arguments or conflict with a partner or spouse	94 (38.4)
	Had to spend a lot more time taking care of a family member	68 (27.8)
**Social activities**		
	Family celebrations canceled or restricted	235 (95.9)
	Separated from family or close friends	218 (89.0)
**Economic activities**		
	Unable to pay important bills like rent or utilities	43 (17.6)
	Difficulty getting places due to less access to public transportation or concerns about safety	33 (13.5)
**Emotional health and well-being**		
	Spent more time on screens and devices (eg, looking at phone, playing video games, and watching TV)	228 (93.1)
	Increase in mental health problems or symptoms (eg, mood, anxiety, and stress)	223 (91.0)
**Physical health problems**		
	More time sitting down or being sedentary	227 (92.7)
	Less physical activity or exercise	206 (84.1)
**Physical distancing and quarantine**		
	Isolated or quarantined due to possible exposure to this disease	134 (54.7)
	Limited physical closeness with child or loved one due to concerns of infection	130 (53.1)
**Infection history**		
	Death of close friend or family member from this disease	41 (16.7)
	Had symptoms of this disease but was never tested	18 (7.3)
**Positive change**		
	More appreciative of things usually taken for granted	192 (78.4)
	More quality time with family or friends in person or from a distance (eg, on the phone, email, social media, video conferencing, and online gaming)	141 (57.6)

^a^The top two indicators in each subcategory with the highest percentage of respondents reporting yes, me or yes, person in home are listed.

^b^Values reported are the number of respondents combined who answered yes, me or yes, person in home.

## Discussion

Overall, in this study, we showed that people who have previously sought mental health treatment reported many negative indicators associated with the COVID-19 pandemic. The least affected age groups included individuals who were 75 years or older and 65 to 75 years old. People with children under the age of 18 years living in the household also reported both more negative indicators and more positive indicators than those without minors living in the home. A marginal effect of gender was noted, but these results should be carefully interpreted, as the sample was not absolutely representative of the population. No effect of mental health diagnosis was noted. Furthermore, this study demonstrates the utility of Grasso and colleagues’ [8] EPII survey in measuring the impacts of epidemics and pandemics in personal and social life domains. Surveys such as this one can help us determine which groups of patients are most at risk of experiencing deleterious effects during stressful situations, such as pandemic lockdowns.

The results of this study are largely in line with other recent findings. For instance, other survey-based research during the COVID-19 pandemic has also shown that older adults appeared to have a more optimistic outlook and better mental health during the early stages of the pandemic [10]. Similarly, other work has shown that younger age predicted more concerns about the threat of COVID-19 across multiple life domains, lower positive affect, higher negative affect, and less frequent positive events [11]. Although we did not see any notable differences in the answers of respondents who completed the survey before, during, or after the stay-at-home orders in the state of California, one study conducted in New Zealand did, in fact, find that the countrywide lockdown had a significant psychological toll on a demographically representative sample of 2010 adult New Zealanders [12].

Our finding that people with minor children living at home experienced more negative indicators is also in line with other recent studies. For example, one survey of parents in the United States during the pandemic found that 27% of parents reported worsening mental health for themselves, and 14% reported worsening behavioral health for their children; further, this worsening of mental health for parents occurred alongside worsening behavioral health for children in nearly 10% of families, among whom 48% reported loss of regular child care, 16% reported change in insurance status, and 11% reported worsening food security due to the pandemic [13]. However, interestingly, these same individuals with children living at home also reported more positive indicators. Taken together with the results of our study, future policy decisions during pandemics, as well as decisions made by mental health practitioners, should keep in mind the special needs of families, particularly those with young children.

Other researchers have also observed, such as we did, that substance use increased during the COVID-19 pandemic, as did other negative mental health outcomes. In one survey of Americans, 40.9% of respondents reported at least one adverse mental or behavioral health condition, including symptoms of anxiety disorder or depressive disorder (30.9%), symptoms of a trauma- and stressor-related disorder related to the pandemic (26.3%), and having started or increased substance use to cope with stress or emotions related to COVID-19 (13.3%) [14].

The data collected here may serve as foundational research in the prevention, care, and treatment of mental health disorders during pandemics such as COVID-19. Dissemination of this new tool, the EPII survey, may be a useful way of measuring the impacts of epidemic- and pandemic-level events not only in this country, but across the world. Understanding how individuals, particularly those individuals at risk due to mental illness, are impacted by events such as COVID-19 may be helpful for determining ways to mitigate the effects of this stress. Further dissemination of this knowledge could be achieved through additional papers, such as this one, that utilize the EPII and presentations or posters at mental health conferences. Without a doubt, more can be done.

In conclusion, we found that people with children under the age of 18 years were most affected by the pandemic. Older age also seemed to be associated with fewer indications of experiencing negative impacts of COVID-19. Gender queer, nonconforming, and transgender individuals may also be at higher risk for negative impacts of COVID-19. No effect of mental health diagnosis was noted. Substance use also tended to increase during the pandemic. Finally, the EPII survey can indeed assess tangible impacts of epidemics and pandemics across personal and social life domains, and it may be a useful tool for future studies that aim to examine the impact of stressful situations on at-risk populations.

## Abbreviations

ANOVA: analysis of variance
EPII: Epidemic-Pandemic Impacts Inventory
ICF: informed consent form
IRB: Institutional Review Board
N/A: not applicable
SAMHSA: Substance Abuse and Mental Health Services Administration
SEM: standard error of the mean
